# Case Report: A case of subarachnoid hemorrhage secondary to elbow intravenous injection of diquat poisoning in young male

**DOI:** 10.3389/fpubh.2025.1729214

**Published:** 2026-02-19

**Authors:** Lingli Song, Wei Fang, Hai Li

**Affiliations:** 1 Department of Emergency, Cheeloo College of Medicine, Weihai Municipal Hospital, Shandong University, Weihai, Shandong, China; 2 Department of Pharmacy, Cheeloo College of Medicine, Weihai Municipal Hospital, Shandong University, WeiHai, Shandong, China; 3 Department of Emergency, Zibo Central Hospital, Zibo, Shandong, China

**Keywords:** acute toxicity, central nervous system injury, diquat poisoning, intravenous injection, subarachnoid hemorrhage

## Abstract

**Background:**

With the prohibition of paraquat, the use of diquat (DQ) as a substitute herbicide has increased significantly, and the number of poisoning cases has also increased. As a highly toxic herbicide, DQ has a high fatality rate. Currently, there is no specific antidote for its poisoning, posing a serious threat to public health.

**Case presentation:**

This paper reports a case of a 23-year-old male patient who self-injected about 120 mL of DQ (20 g/100 mL) by elbow vein injection due to emotional excitement 1 h before admission. Nausea, vomiting, and pain occurred 10 min after injection. After admission, emergency head CT showed subarachnoid hemorrhage (SAH) and elevated blood pressure to 207/115 mmHg. Although the patient was admitted to the emergency intensive care unit (EICU) of our hospital for further treatment and given symptomatic treatment such as blood purification, massive rehydration, promotion of excretion, anti-oxidation, protection of organ function, and sedation as soon as possible, the patient still died of respiratory and circulatory failure 6 h after admission. Liquid chromatography–tandem mass spectrometry (LC–MS/MS) results showed that the DQ concentrations were 31.10 μg/mL in plasma and 147.60 μg/mL in urine.

**Conclusion:**

This case suggests that a massive dose of intravenous DQ poisoning can lead to severe central nervous system injury and rapid death. Based on the clinical presentation and existing literature, the mechanism may be related to the extremely high concentration of DQ rapidly crossing the blood–brain barrier and entering brain tissue, thereby causing tissue cell damage or reduction–oxidation (redox) cycle. This highlights that we need to be highly vigilant against the clinical hazards caused by the unconventional poisoning pathway of DQ.

## Introduction

Following the prohibition of paraquat (PQ), the use of diquat (DQ) as a substitute herbicide has increased significantly, leading to a rise in DQ poisoning cases in recent years (1). DQ is a non-selective bipyridyl herbicide that acts by disrupting cell membranes and interfering with photosynthesis (2) Its toxicity in humans is primarily mediated by the generation of reactive oxygen species (ROS) through a redox cycling process, leading to oxidative stress, lipid peroxidation, mitochondrial damage, and ultimately cell death (3). As a result, DQ poisoning poses a significant public health problem. While the reported mortality rate in the United States is relatively low (< 3%), the situation in China is far more severe, with mortality rates ranging from 16.7% to as high as 60.0%, primarily due to intentional ingestion for suicidal purposes (4).

DQ can enter the body through the digestive tract, respiratory tract, eye or skin mucous membrane contact, resulting in poisoning. Its primary target organs include the kidneys, lungs, liver, heart, and central nervous system, potentially causing multi-organ dysfunction and death (5, 6). At present, there is no specific antidote for DQ poisoning. The mainstay of treatment involves supportive care, including gastrointestinal decontamination to reduce absorption, enhanced elimination via hemoperfusion and hemofiltration, and antioxidant therapy to mitigate cellular damage (3). Due to multiple organ involvement, patients can have a variety of manifestations, indicating the need for more effective treatment options (7).

Oral ingestion is the most common route of poisoning in clinical practice. It has been reported that drug poisoning can be caused by respiratory tract and skin mucosal contact absorption, intramuscular injection, subcutaneous injection and vaginal contact (5, 8, 9). However, the intravenous route is exceptionally rare. To the best of our knowledge, after a systematic search of PubMed and Cochrane Library databases using the terms “diquat,” “intravenous,” “poisoning” and “hemorrhage,” up to November 2025, this appears to be the first reported case of subarachnoid hemorrhage secondary to a high-dose intravenous injection of DQ. This paper reports the first case of subarachnoid hemorrhage (SAH) secondary to high-dose DQ poisoning by cubital vein injection in Weihai Municipal Hospital Affiliated to Shandong University. This report highlights the serious damage caused by intravenous DQ poisoning to the patient’s nervous system, and enriches the treatment experience of DQ rapid unconventional poisoning pathway.

## Case description

On February 21, 2024, a previously healthy 23-year-old male worker (height: 170 cm, weight: 65 kg) self-injected approximately 120 mL (20 g/100 mL) of DQ via the elbow vein due to emotional agitation. Ten minutes after administration, the patient began to experience headache, nausea, and vomiting of gastric contents. He reported generalized body pain and irritability, but no fever, sweating, syncope, episodes of transient visual loss (amaurosis), diarrhea, or constipation. The referring hospital did not provide any specific treatment as the patient’s respiratory and circulatory functions were relatively stable, and transferred him directly to our institution. He arrived at our hospital’s emergency department at 17:00. This medical history was provided by the patient himself.

**Figure 1:**
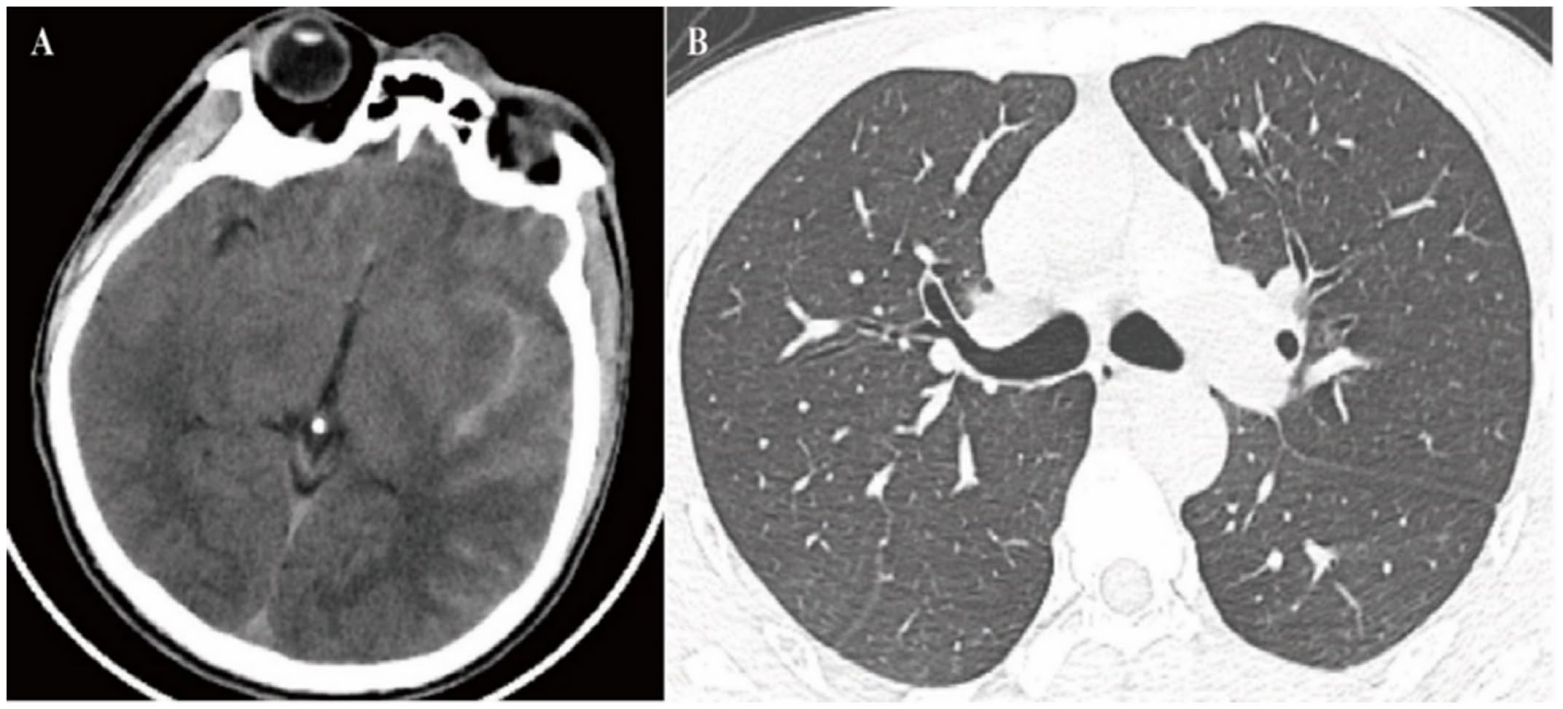
CT imaging findings. **(A)** Non-contrast head CT showing high-density shadows in the suprasellar cistern and lateral fissure cistern, consistent with SAH. **(B)** Chest CT demonstrating scattered ground-glass opacities and multiple nodules in both lungs, suggestive of inflammatory exudation.

On admission, blood pressure was 207/115 mmHg, heart rate was 78 beats per minute, and blood oxygen saturation 99%. The patient is conscious but agitated. Physical examination of the injection site at the elbow revealed a clear puncture mark without significant swelling, hematoma, or signs of extravasation, suggesting the injection was likely administered entirely intravenously. His breath sounds were clear in both lungs, and the abdomen was soft with diffuse tenderness but no rebound tenderness. Emergency blood routine test results (Table 1) showed leukocytosis (12.26 × 10^9^/L) with marked lymphocytosis (4.26 × 10^9^/L) and mild neutrophilia (7.23 × 10^9^/L), consistent with the hematological pattern of inflammation and immune activation in early-stage DQ poisoning. Lactate dehydrogenase (LDH), *α*-hydroxybutyrate dehydrogenase (α-HBDH), and creatine kinase MB isoenzymes were significantly elevated, suggestive of myocardial injury. In addition, emergency computed tomography (CT) of the head revealed high-density shadows in the suprasellar and lateral fissure cisterns, consistent with subarachnoid hemorrhage (SAH) (Figure 1A); chest CT showed scattered ground-glass shadows and multiple nodules in both lungs, suggesting inflammatory exudation (Figure 1B); no parenchymal organ damage was found in the abdomen. For further treatment, he was admitted to the emergency intensive care unit (EICU) of our hospital at 17:40, and was treated with omeprazole to inhibit acid and protect the stomach, a large amount of rehydration, glutathione, acetylcysteine antioxidant and other treatments.

**Table 1:** Consolidated laboratory and blood gas analysis results.

Category	Index	Normal range	17:00	17:55	20:00	22:30
Hematology	WBC (×10^9^/L)	3.50–9.50	12.26		24.69	
	NEUT (×10^9^/L)	1.8–6.30	7.23		22.56	
	LYMPH (×10^9^/L)	1.10–3.20	4.26			
	LYMPH (%)	20.00–50.00			7.80	
	RBC (×10^12^/L)	3.80–5.10			4.23	
	Hb (g/L)	130.00–175.00		157.00	132.00	139.00
	HCT (%)	40.00–50.00			38.70	
	PLT (×10^9^/L)	125.00–350.00			96.00	
Blood gas	pH	7.35–7.45		7.06	7.30	7.27
	PaCO₂ (mmHg)	35.00–45.00		33.90	31.40	35.20
	PaO₂ (mmHg)	80.00–108.00		143.00	220.10	225.00
	K^+^ (mmol/L)	3.40–4.50		2.90	3.79	3.10
	Na^+^ (mmol/L)	135.00–145.00		150.00	133.90	
	Cl^−^ (mmol/L)	98.00–107.00		116.00	105.70	
	Ca^2+^ (mmol/L)	1.15–1.29		1.28	0.95	
	Lac (mmol/L)	0.50–1.60		18.00	12.80	11.50
	HCO₃^−^ (mmol/L)	21.00–25.00		9.60	14.90	16.30
	BE (mmol/L)	−3.0-3.0		−19.80	−10.31	−9.70
	AG (mmol/L)	8.00–16.00		24.60	19.45	17.70
	Glu (mmol/L)	3.90–6.10		15.00	15.74	6.90
	PaO₂/FiO₂ (mmHg)	400.00–500.00				563.00
Biochemistry	K (mmol/L)	3.50–5.30			3.84	
	Na (mmol/L)	137.00–147.00			136.80	
	Cl (mmol/L)	99.00–110.00			100.80	
	Ca (mmol/L)	2.11–2.52			1.97	
	CO₂CP (mmol/L)	21.00–31.00			16.55	
	Unconjugated Bilirubin (mmol/L)	1.50–11.10	12.00			
	LDH (U/L)	120.00–250.00	347.43		606.53	
	α-HBDH (U/L)	72.00–182.00	308.00		437.00	
	CK (U/L)	40.00–200.00			224.23	
	CK-MB (U/L)	< 25	38.76		104.13	
	cTnI (ng/mL)	< 0.014			1.944	
	AST (U/L)	13–35.00			308.25	
	ALT (U/L)	7.00–40.00			94.55	
	Cr (μmol/L)	41.00–81.00			153.88	
	BUN (mmol/L)	3.10–8.80			4.35	
	UA (μmol/L)	142.80–339.20			647.90	
	BHB (mmol/L)	0.03–0.3	0.54			

WBC, white blood cell; NEU, neutrophil; LYMPH, lymphocyte; RBC, red blood cells; Hb, hemoglobin; HCT, hematocrit; PLT, platelets; pH, potential of Hydrogen; PaCO₂, partial pressure of carbon dioxide; PaO_2_, partial pressure of oxygen; HCO_3_^−^, bicarbonate; BE, base excess; Lac, lactate; AG, anion gap; K^+^, serum potassium; Na^+^, serum sodium; Ca^*2*+^, serum calcium; Cl^−^, serum chloride; Glu, Glucose; BUN, Blood Urea Nitrogen; Cr, Creatinine; UA, Uric Acid; CO_2_CP, carbon dioxide combining power; BHB, β-hydroxybutyric acid; LDH, lactate dehydrogenase; α-HBDH, α-hydroxybutyrate dehydrogenase; CK, creatine kinase; CK-MB, creatine kinase-MB; cTnI, cardiac troponin I; ALT, alanine aminotransferase; AST, aspartate aminotransferase; PT, prothrombin time.

At 17:55, the patient developed epilepsy and was sedated with diazepam. Simultaneously administered endotracheal intubation and mechanical ventilation treatment. Subsequently, blood gas analysis was performed (Table 1), the results showed that severe high anion interstitial metabolic acidosis (pH 7.06, HCO₃^−^ 9.6 mmol/L, base excess −19.8 mmol/L, anion gap 24.6 mmol/L), markers of end-organ hypoperfusion (lactate 18 mmol/L), and characteristic electrolyte derangements of diquat poisoning including hypokalemia (2.9 mmol/L) and hypocalcemia (1.28 mmol/L). These results are consistent with the end-stage metabolic disorder of diquat poisoning, indicating profound collapse of whole-body cellular energy metabolism. At 18:00, right femoral vein puncture was performed for catheter placement, followed by continuous venous hemofiltration (CVVH) combined with hemoperfusion treatment without heparinization.

At 20:00, repeat laboratory tests were conducted. The results of coagulation function showed that the coagulation function was disordered, and disseminated intravascular coagulation may occur (PT 14.7 s, PT activity 68%, fibrinogen 1.29 g/L, D-dimer 26.11 mg/L), which may be the result of DQ directly damaging the vascular endothelium, activating the coagulation cascade, and causing microthrombosis and secondary hyperfibrinolysis. The results of gas analysis and blood routine showed that the patient had severe metabolic acidosis (pH 7.30, HCO₃^−^ 14.90 mmol/L, base excess −10.31 mmol/L) with profound lactic acidosis (lactate 12.80 mmol/L), widened anion gap (19.45 mmol/L), and systemic inflammatory response. Biochemical results confirmed multiorgan dysfunction including myocardial injury (cTnI 1.944 ng/mL, CK-MB 104.13 U/L), hepatocellular injury (AST 308.25 U/L, ALT 94.55 U/L), and acute kidney injury (Cr 153.88 μmol/L). Despite treatment of status epilepticus with repeated diazepam and propofol, seizure control remained inadequate. After emergency consultation in neurology and neurosurgery, mannitol dehydration and nimodipine anti-cerebrovascular spasm treatment were added, yet the patient’s condition continued to deteriorate.

At 22:30, blood gas analysis showed persistent metabolic acidosis (Table 1). The results of blood gas analysis showed that metabolic acidosis continued to deteriorate. Although lactate levels decreased to 11.5 mmol/L and anion gap narrowed to 17.7 mmol/L, Insufficient respiratory compensation (PCO₂ 35.2 mmHg) leads to the progressive aggravation of acidemia. New-onset severe hypokalemia (K^+^ 3.1 mmol/L) and rapidly decreasing blood glucose (6.9 mmol/L) indicated emergent risks of treatment-induced electrolyte and metabolic derangements. Tissue oxygen utilization disorder persisted, characterized by the dissociation of hyperlactacidemia (>10 mmol/L) and a normal oxygenation index (563 mmHg) (Table 1), indicating that DQ poisoning-induced mitochondrial toxicity and multi-organ damage were still progressing.

**Figure 2:**
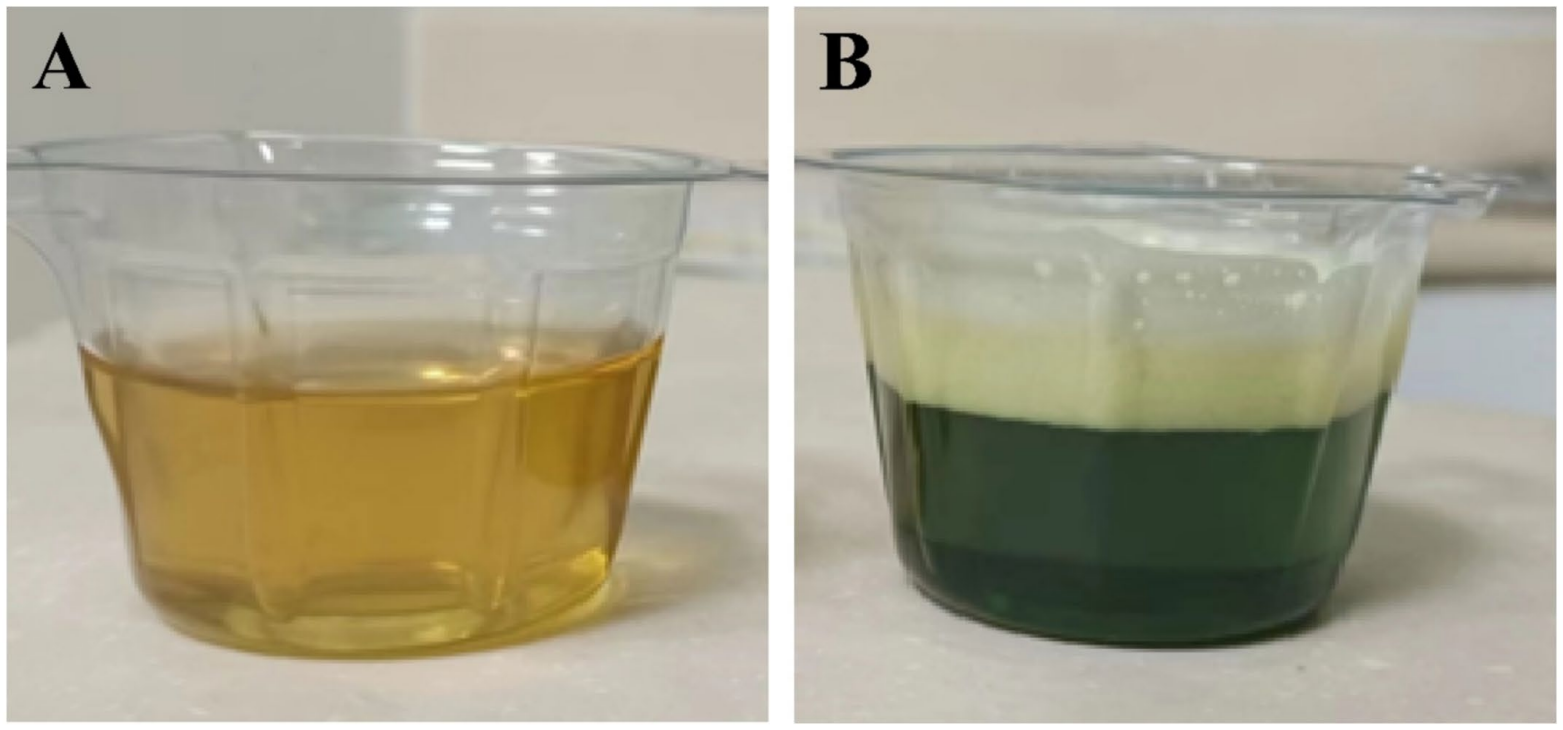
Images of urine for the 23-year-old man who self-injected ~120 mL of diquat. **(A)** The patient’s original urine. **(B)** The patient’s urine turned dark green after the addition of sodium dithionite.

At 00:00, the patient’s blood pressure fell to 62/28 mmHg, and norepinephrine and other pressor drugs were applied and actively rescued. Finally, at 01:21 on February 22, his heart stopped and he was declared clinically dead. The patient’s original urine is shown in Figure 2A, was collected upon admission. The sample underwent bicarbonate/sodium dithionite testing at around 17:30, and the urine appeared dark green (Figure 2B). Subsequently, the concentration of hematuria was detected by liquid chromatography tandem mass spectrometry. LC–MS/MS results showed that the DQ concentrations were 31.10 μg/mL in plasma and 147.60 μg/mL in urine.

## Discussion

As an alternative herbicide to PQ, the poisoning mechanism and target organ damage characteristics of DQ have gradually become a focus of clinical research. To the best of our knowledge, this is the first reported case of a fatal subarachnoid hemorrhage following a massive intravenous injection of DQ. A systematic literature search was conducted in PubMed and Embase using the keywords “diquat,” “intravenous,” “poisoning,” and “hemorrhage,” which yielded no similar cases. This case is unique due to the direct entry of a massive dose of the toxin into the systemic circulation, bypassing the first-pass metabolism that typically occurs with oral ingestion. The patient received a direct injection of 24 g DQ, resulting in a peak blood concentration of 31.10 μg/mL within 1 h, a level significantly higher than the reported lethal blood concentrations in oral poisoning cases, which are often in the range of 0.3–2.0 μg/mL (10, 11)

The clinical presentation of this case differs markedly from typical oral DQ poisoning. In oral cases, gastrointestinal symptoms are prominent initially, followed by a gradual onset of multi-organ damage, particularly acute kidney injury and lung fibrosis, over several days (3, 12). Catastrophic central nervous system (CNS) toxicity, while reported, is often a later complication. In contrast, this intravenous case presented with immediate and dominant neurological catastrophe, a toxicokinetic profile that more closely resembles a massive, direct vascular insult. This is consistent with other reports of parenteral DQ administration, which also show accelerated and severe systemic toxicity compared to oral exposure (9).

The development of SAH in this patient is likely multifactorial. First, the massive dose and high concentration of DQ likely caused direct endothelial damage to the cerebral vasculature, increasing its permeability. The intense oxidative stress from DQ’s redox cycling can disrupt the tight junctions of the BBB, facilitating toxin entry and causing vasogenic edema and hemorrhage (13, 14). Furthermore, the patient’s extremely high blood pressure (207/115 mmHg) on admission, a state of hypertensive crisis, likely served as a significant trigger for the SAH, especially in the context of toxin-induced endothelial damage. Cases of DQ poisoning complicated by intracerebral bleeding have been reported, supporting the link between DQ toxicity and cerebrovascular events (15).

The qualitative urine test using sodium bicarbonate and sodium dithionite is a rapid bedside tool for detecting bipyridyl herbicides. The principle is that in an alkaline environment, sodium dithionite reduces diquat, producing a green-colored free radical, confirming its presence (3). While this test is useful for rapid diagnosis, the dark green color observed in this patient’s urine qualitatively indicated a very high concentration, which was later confirmed by quantitative analysis.

Current management guidelines for DQ poisoning, primarily based on oral ingestion cases, focus on preventing absorption (gastric lavage, activated charcoal) and enhancing elimination (forced diuresis, hemoperfusion) (16). While this patient received aggressive treatment, including immediate hemoperfusion and CVVH, these measures were insufficient to counteract the overwhelming toxic load delivered directly into the circulation. The rapid onset of irreversible CNS damage and multi-organ failure suggests that by the time treatment was initiated, the cellular damage was already too extensive. This case underscores the profound limitations of current therapies in the face of massive intravenous exposure.

From a public health perspective, this case is a stark reminder of the dangers posed by highly toxic herbicides, particularly as DQ becomes more widely used following the ban on paraquat. The accessibility of such a lethal substance, especially to individuals in emotional distress, is a major concern. This highlights the need for stricter regulations on the sale and storage of DQ, public awareness campaigns about its extreme toxicity regardless of the exposure route, and improved mental health support in rural and agricultural communities.

This report has several limitations. As a single case report, it is difficult to generalize the findings. We were unable to obtain serial blood DQ concentrations to perform a full toxicokinetic analysis. Furthermore, an autopsy was not performed, which limits our ability to pathologically confirm the extent of CNS and other organ damage. Future research should focus on animal models of intravenous DQ poisoning to better understand the pathophysiology and to test potential neuroprotective therapies. More case reports, if they emerge, would be valuable for building a more complete clinical picture.

## Conclusion

This case report documents a rare and fatal outcome of a massive intravenous injection of diquat, characterized by rapid-onset subarachnoid hemorrhage and multi-organ failure. Our findings underscore the extreme toxicity of DQ when administered parenterally, leading to a clinical trajectory markedly different from typical oral ingestion cases.

The increasing use of DQ as a substitute for paraquat presents a significant and ongoing public health concern. This case serves as a stark reminder of the lethal potential of DQ, particularly its accessibility to vulnerable individuals. It highlights the urgent need for stricter regulations on the sale and distribution of highly toxic herbicides and for greater public awareness of their dangers, irrespective of the route of exposure. Furthermore, the catastrophic outcome, despite aggressive and timely medical intervention including hemoperfusion, exposes the profound limitations of current treatment modalities in managing massive, direct circulatory poisoning.

Future research is imperative to better understand the pathophysiology of intravenous DQ poisoning. Animal models are needed to investigate the specific mechanisms of blood–brain barrier disruption and CNS injury. Additionally, there is a critical need to develop and evaluate novel therapeutic strategies, such as targeted neuroprotective agents or more effective detoxification methods, to improve outcomes in these severe poisoning scenarios. A deeper understanding of the unique challenges posed by unconventional poisoning routes is essential for advancing clinical practice and public health policy.

## Data Availability

The raw data supporting the conclusions of this article will be made available by the authors, without undue reservation.
